# TWIST1 induces MMP3 expression through up-regulating DNA hydroxymethylation and promotes catabolic responses in human chondrocytes

**DOI:** 10.1038/srep42990

**Published:** 2017-02-21

**Authors:** Joe Hasei, Takeshi Teramura, Toshiyuki Takehara, Yuta Onodera, Takuro Horii, Merissa Olmer, Izuho Hatada, Kanji Fukuda, Toshifumi Ozaki, Martin K. Lotz, Hiroshi Asahara

**Affiliations:** 1 Department of Molecular and Experimental Medicine, The Scripps Research Institute, La Jolla, CA, USA; 2 Division of Cell Biology for Regenerative Medicine, Institute of Advanced Clinical Medicine, Kindai University, Faculty of Medicine, Osaka, Japan; 3 Laboratory of Genome Science, Biosignal Genome Resource Center, Institute for Molecular and Cellular Regulation, Gunma University, Gunma, Japan; 4 Department of Rehabilitation Medicine, Kindai University, Faculty of Medicine, Osaka, Japan; 5 Science of Functional Recovery and Reconstruction, Okayama University Graduate School of Medicine, Dentistry and Pharmaceutical Sciences, Okayama, Japan; 6 Department of Systems BioMedicine, Graduate School of Medical and Dental Sciences, Tokyo Medical and Dental University, Tokyo, Japan

## Abstract

The objective was to investigate the levels of TWIST1 in normal and OA cartilage and examine its role in regulating gene expression in chondrocytes. Human cartilage tissues and chondrocytes were obtained at autopsy from normal knee joints and from OA-affected joints at the time of total knee arthroplasty. TWIST1 expression was increased in human OA knee cartilage compared to normal knee cartilage. TWIST1 induced matrix metalloproteinase 3 (MMP3) expression without direct binding to MMP3 promoter and increased the 5-hydroxymethylcytosine (5hmC) level at the MMP3 promoter. The effect of TWIST1 on expression of TET family (TET1, 2 and 3) was measured in stable TWIST1 transfected TC28 cells, and TET1 expression was up-regulated. TWIST1 dependent upregulation of Mmp3 expression was suppressed in Tet triple KO fibroblast derived from mouse ES cells. Increased TWIST1 expression is a feature of OA-affected cartilage. We identified a novel mechanism of catabolic reaction where TWIST1 up-regulates MMP3 expression by enriching 5hmC levels at the MMP3 promoter via TET1 induction. These findings implicate TWIST1 as an important factor regulating OA related gene expression. Clarifying epigenetic mechanisms of 5hmC induced by TWIST1 is a critical molecule to understanding OA pathogenesis.

Dysregulated chondrocyte activation and de-differentiation in articular cartilage are important factors in Osteoarthritis (OA) pathogenesis^1^,^2^. Twist-related protein1 (TWIST1) is known as Class A basic helix-loop-helix protein 38 (bHLHa38), and necessary for the embryonic development through epithelial-mesenchymal transitions (EMT), and regulates the formation of mesoderm and morphogenesis^3^,^4^,^5^,^6^. TWIST1 regulates cellular determination and differentiation of several lineages including myogenesis and osteogenesis^7^. A critical role in mesoderm development is indicated by the association of TWIST1 gene mutations with Saethre-Chotzen syndrome, characterized by a broad range of congenital anomalies, including fusion of the skin between the second and third fingers, short stature, craniosynostosis, head and facial asymmetry and ptosis^8^,^9^. Twist1 is expressed in immature chondrocytes, but decreases in mature chondrocytes^10^. Twist1 is known not only as a suppresser of Runx2 which is necessary for osteoblast differentiation and bone formation, but also as a regulator of mesenchymal stem cell differentiation into chondrocytes^11^. Twist1 interferes with chondrocyte gene expression through Wnt and BMP-dependent signaling^10^. Twist1 also binds to the 3′UTR and the HMG DNA-binding domain of Sox9, a master regulator of chondrogenesis, and suppresses Sox9-dependent gene activation^12^,^13^. However, expression of TWIST1 in mature normal and OA cartilage and its effects in mature chondrocytes are unknown.

Recently, Twist1 was also identified as a key factor of cancer metastasis and stemness through EMT^14^,^15^, correlating tumor invasion with MMP1, MMP2 and MMP9 regulation^16^,^17^. MMPs are classified based on substrate specificity (Collagenase (MMP1, 8 and 13), Gelatinase (MMP2 and 9), Stromelysin (MMP3, 10 and 11)), and mediate cartilage degradation in OA^18^,^19^,^20^. In this regard, we were interested in the potential link between TWIST1 and MMPs in human chondrocytes and OA pathogenesis.

Cytosine methylation changes the interactions between proteins and DNA, and methylation changes at CpG sites in promoter regions have been suggested to mediate OA related gene expression^21^. In OA chondrocytes, some MMP promoters show decreased methylation at CpG loci compared to normal chondrocytes^22^. Recently, 5-hydroxymethylcytosine (5hmC) has been identified as a new constituent of mammalian DNA, and recognized as the sixth base. Conversion of 5mC to 5hmC by TET (ten eleven translocation) family of Fe(II) and 2-oxoglutarate-dependent enzymes facilitates DNA demethylation, and regulates gene expression^23^,^24^,^25^. The 5hmC levels were enriched in the central nervous system, and play important roles in embryonic stem cells^26^,^27^. In OA chondrocytes, a global increase of 5hmC and increased locus specific 5hmC at CpG sites in the MMP1 and MMP3 were reported compared with normal chondrocytes^28^. However, the mechanisms leading to 5hmC changes in chondrocytes are largely unknown.

Here, we found that TWIST1 was upregulated in human OA knee cartilage tissue compared with normal knee cartilage. In this study, we focused on the relationship between TWIST1 and MMP expression through 5hmC status in MMP3 promoter CpG sites.

## Results

### TWIST1 is highly expressed in human OA cartilage

**Figure 1:**
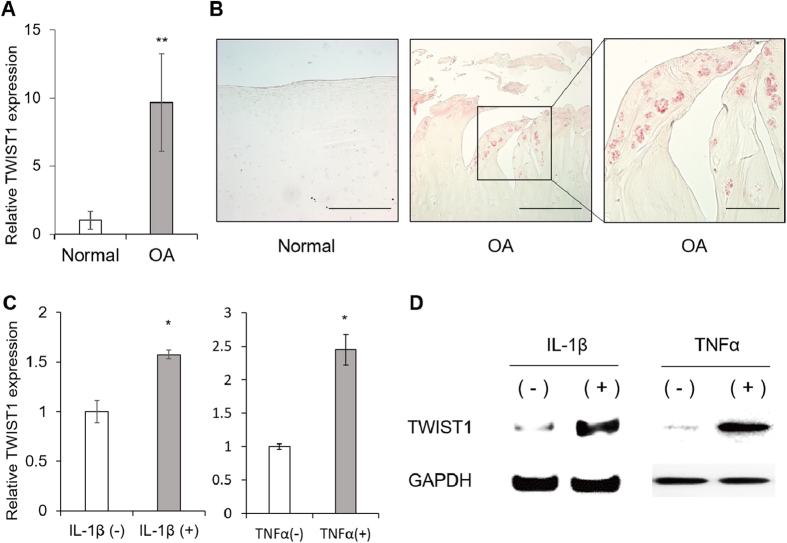
TWIST1 is highly expressed in human OA cartilage. (**A**) TWIST1 gene expression in cartilage tissues from 7 normal donors and 8 OA donors were analyzed by real-time qRT-PCR. TWIST1 gene expression levels in OA cartilage tissues are relative to normal cartilage tissues and normalized to GAPDH. Values are the mean ± SEM ratio. (**B**) Immunohistochemical staining for TWIST1 in human normal and OA cartilage tissue. **P < 0.01. Left scale bars, 500 μm. Right scale bar, 200 μm. (**C**) The TWIST1 expression changes after IL-1β and TNFα stimulation in human primary chondrocyte were confirmed by real-time PCR. Values are the mean ± SEM. *P < 0.05. (**D**) The TWIST1 expression changes after 12 hours IL-1β and TNFα stimulation in human primary chondrocyte were confirmed by Western blotting.

We analyzed TWIST1 expression in cartilage by real-time qRT-PCR and IHC. TWIST1 mRNA levels were significantly increased in human OA knee cartilages compared to normal knee cartilages (approximately 10-fold up-regulation) (Fig. 1A). By IHC, TWIST1 positive cells were observed in human OA cartilage tissues, mainly in chondrocyte clusters, but few in normal cartilage regions (Fig. 1B). The TWIST1 expression was up-regulated after IL-1β (interleukin-1β) and TNFα (tumor necrosis factor α) stimulation (Fig. 1C,D).

### TWIST1 induces MMP3 expression

**Figure 2:**
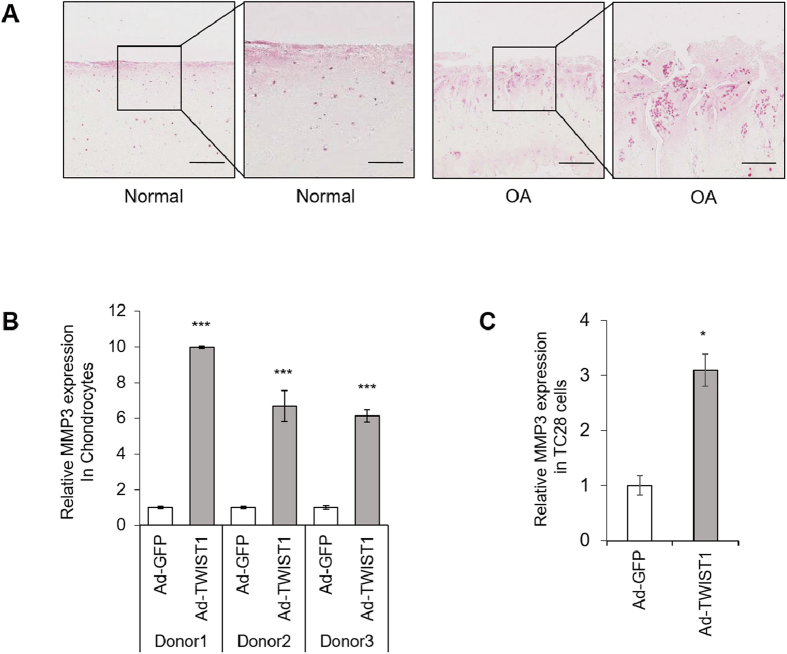
TWIST1 regulates MMP3 expression. (**A**) Immunohistochemistry analysis of human cartilage tissues. Cartilage sections were obtained from normal and OA-affected cartilages. Sections were stained with MMP3 antibody. Left scale bars, 500 μm. Right scale bar, 200 μm. (**B**) MMP3 expression was detected by real-time qRT-PCR in human chondrocytes from 3 donors after Ad-GFP or Ad-TWIST1 infection at 72 hours. Values are the mean ± SEM of three independent experiments for each donor. (**C**) Relative MMP3 expression level in TC28 after Ad-GFP or Ad-TWIST1 infection. Values are the mean ± SEM. *P < 0.05, ***P < 0.001.

**Figure 3:**
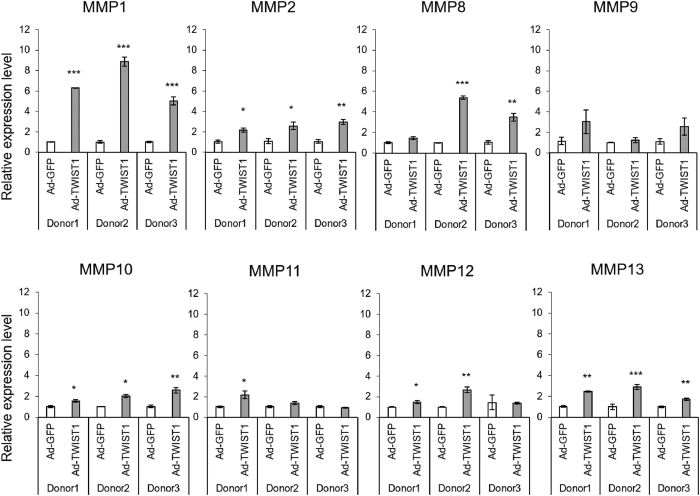
MMP expression in human chondrocytes after Ad-TWIST1 infection. Normal human chondrocytes from 3 donors were infected Ad-GFP or Ad-TWIST1 for 72 hours. MMP expression was analyzed by real-time qRT-PCR. Values are the mean ± SEM of three independent experiments for each donor. *P < 0.05, **P < 0.01, ***P < 0.001.

Matrix metalloproteinases are major factors contributing joint tissue destruction^29^. Immunohistochemistry showed, in OA cartilage, MMP3 is highly expressed, similar to TWIST1 (Fig. 2A). This prompted us to test whether TWIST1 would be a regulator of MMPs in chondrocytes. We examined MMP gene expression by real-time qRT-PCR in human primary chondrocytes following Ad-TWIST1 infection compared with Ad-GFP infection. MMP3 was significantly up-regulated by Ad-TWIST1 (5–10 fold change) (Fig. 2B). In TC28 cells, MMP3 mRNA level was also increased by Ad-TWIST1 infection by approximately 3 times (Fig. 2C). Ad-TWIST1 up-regulated almost all MMPs, but MMP1 and MMP3 increased much more compared with MMP2, 8, 9, 10, 11, 12 and 13 in human primary chondrocytes (Fig. 3).

### Methylation status of CpG loci in MMP3 promoter

**Figure 4:**
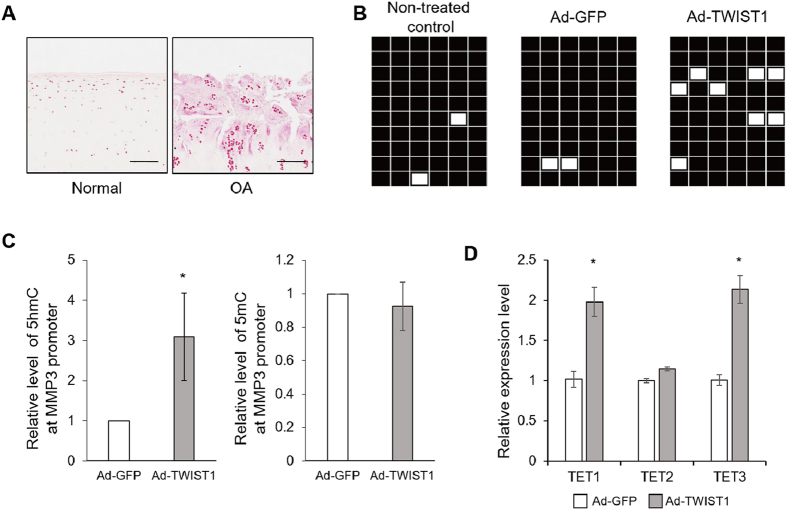
DNA methylation and 5hmC status in the MMP3 promoter. (**A**) Immunohistochemistry analysis of human cartilage tissues. Cartilage sections were obtained from normal and OA-affected cartilages. Sections were stained with 5hmC anti body. Left scale bars, 500 μm. Right scale bar, 200 μm. (**B**) Bisulfite genomic sequencing results of the MMP3 promoter regions in TC28 cells after Ad-GFP or Ad-TWIST1 infection at 72 hours. Black, methylated; white, unmethylated. (**C**) To detect locus-specific 5hmC and 5mC status at CCGG site in MMP3 promoter, restriction enzyme digestion and quantitative polymerase chain reaction were performed. *P < 0.05. (**D**) TET1, TET2, and TET3 expressions were detected by real-time qRT-PCR in human chondrocytes after Ad-GFP or Ad-TWIST1 infection at 72 hours. Values are the mean ± SEM of three independent experiments. *P < 0.05.

We confirmed 5hmC enrichment in human OA cartilage by immunohistochemistry (Fig. 4A). In TC28 cells, a bisulfite DNA sequencing analysis showed the MMP3 promoter CpG loci were methylated, and Ad-TWIST1 didn’t affect the methylation status compared with non-treated cells (NTC) and Ad-GFP-treated group (Fig. 4B). However, 5hmC status level around a CCGG site in MMP3 promoter was specifically increased, but 5mC status level didn’t change by Ad-TWIST1 compared with Ad-GFP (Fig. 4C). In human chondrocytes, TET1 and TET3 mRNA level were increased by Ad-TWIST1 infection by approximately 2 fold (Fig. 4D). These results suggest TWIST1 promotes 5hmC of specific MMP3 promoter sites through TET1 and TET3 up-regulation.

### TWIST1 regulates TET1 expression in TC28 cells

**Figure 5:**
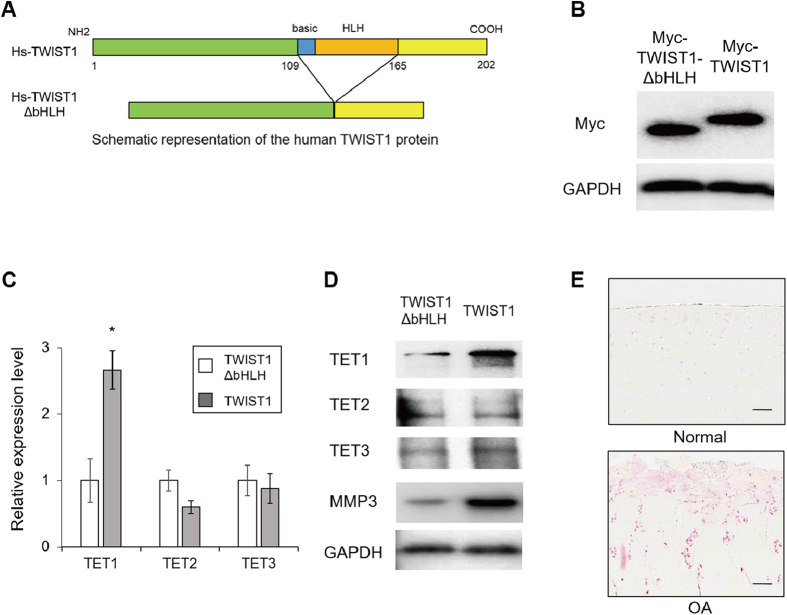
Effect of TWIST1 on TET1 expression in TC28 cells. (**A**) Schematic representation of the human TWIST1 protein. (**B**) Myc-tagged TWIST1 or TWIST1-ΔbHLH stably transfected TC28 cells were generated. Myc expression was analyzed by Western blotting. (**C**) TET1, 2, and 3 gene expression levels in TWIST1 stably transfected TC28 cells (TC28-TWIST1) were determined by quantitative PCR. Stable TWIST1-ΔHLH transfected TC28 cells (TC28-TWIST1-ΔHLH) were used as control. (**D**) Western blot analysis of TET 1, 2 and 3 expression in TC28-TWIST1 and TC28-TWIST1-ΔHLH cells. All gels for western blot analysis were run under the same experimental conditions, and western blot images were cropped with a grey cropping line. (**E**) Immunohistochemistry analysis of human cartilage tissues. Cartilage sections were obtained from normal and OA-affected cartilages. Sections were stained with TET1 anti body. Scale bar, 200 μm.

To further investigate the mechanism of 5hmC enrichment by TWIST1, we generated Myc-tagged TWIST1 stably transfected TC28 cells (Myc-TWIST1-TC28) using piggyBac transposon (Fig. 5A,B). In Myc-TWIST1-TC28 cells TET1 expression was dramatically increased, but there were no differences in TET2 and TET3 in western blot (Fig. 5C,D). This result suggests that TWIST1 increases 5hmC levels at the MMP3 promoter through TET1 up-regulation. Immunohistochemistry showed, in human OA cartilage tissue, TET1 is highly expressed, similar to TWIST1 (Fig. 5E). The TET1 expression increased in both chondrocytes and TC28 cell after TWIST1 overexpression. Therefore we focused on the relationship TET1 and MMP3.

### TET1 suppression by siRNA inhibited MMP3 enhancement by TWIST1 in human chondrocytes

**Figure 6:**
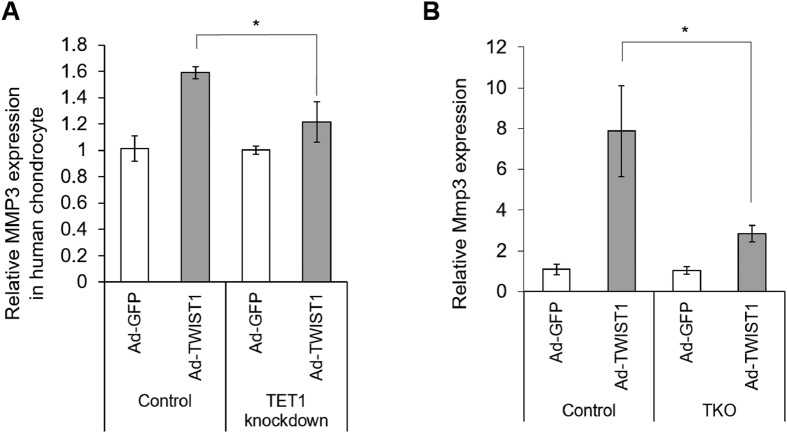
MMP3 expression change after TWIST1 overexpression under TET knock down. (**A**) The siRNA for TET1 was transfected to human primary chondrocytes, and after 36 hours, cells were infected with Ad-GFP or Ad-TWIST1 and cultured for 72 hours. The MMP3 gene expression was determined by real-time qRT-PCR. (**B**) Fibroblasts were derived from Tet1, 2 and 3 triple knock out mice embryonic stem cells, and were transfected Ad-GFP or Ad-TWIST1 for 72 hours. Mmp3 gene expression was determined by quantitative PCR. *P < 0.05.

To test if TET1 plays a critical role in TWIST1 dependent MMP3 upregulation, first we treated human chondrocytes TET1 siRNA for 36 hours, the cells were infected with Ad-TWIST1 and cultured for 72 hours. TWIST1 dependent MMP3 upregulation was suppressed by siTET1 treatment (Fig. 6A).

### TWIST1 does not induce Mmp3 expression in Tet-deficient fibroblasts

To reveal the role of TETs more clearly, we obtained fibroblasts from wild-type or Tet1, 2 and 3 triple knockout mutant embryonic stem cells (ESCs)^30^,^31^. Wild-type and Tet-KO fibroblasts were infected with Ad-GFP or Ad-TWIST1 and cultured for 72 hours. TWIST1 dependent upregulation of Mmp3 expression was markedly inhibited in Tet-KO fibroblast cells (Fig. 6B), suggesting that Tet proteins are essential for driving TWIST1 dependent MMP3 induction.

## Discussion

Some reports showed that Twist1 inhibited transactivator function of master chondrogenic regulator Sox9^12^,^13^, and knockdown of Twist1 transcript levels caused increased expression of cartilage-specific genes, such as aggrecan and type II collagen^10^,^32^. Our data shows that TWIST1 was expressed at much higher levels in OA cartilage compared with normal cartilage, providing the first evidence to reveal a correlation between TWIST1 and OA.

Methylation of DNA in the promoter regions of a target gene is well known to regulate gene expression^33^. In cartilage and chondrocytes, some reports have shown that the DNA methylation status was regulated in individual gene promoters^34^,^35^,^36^. This implicates DNA methylation as a mechanism of OA-related gene expression. For instance, at the COL10A1 promoter, two CpG sites were demethylated during chondrocyte differentiation from mesenchymal stem cells, and correlated with COL10A1 expression^37^. Hypermethylated COL9A1 enhancer was associated with transcriptional repression in OA cartilage^38^, and the SOX9 promoter was hypomethylated during chondrogenesis of human synovium-derived mesenchymal stem cells^39^. Several studies reported DNA methylation changes in OA cartilage^34^,^40^,^41^,^42^,^43^. Jeffries *et al*. performed genome-wide DNA methylation study in cartilage from hip OA patients^44^. They identified 550 differentially methylated sites in OA, and approximately one-third of known OA susceptibility genes showed differential methylation. The major enzymes that mediate the destructive processes in joint tissues in OA MMPs. Roach *et al*. examined the methylation status of the promoter regions of MMP3, MMP9, and MMP13 in OA chondrocytes, and revealed the overall percentage of non-methylated site was increased^22^. We checked MMP3 promoter methylation status by bisulfite sequencing analysis, but the percentage of non-methylated CpG sites did not change after Ad-TWIST1 infection (Fig. 3B). This suggested that TWIST1 regulates MMP3 through other epigenetic mechanisms.

Recently, 5hmC was discovered as a sixth base of the genome, and converted from 5mC by TET family proteins. Many reports showed 5hmC and TET proteins have important roles of epigenetic reprogramming and regulating tissue-specific gene expression. The highest levels of 5hmC were found in the brain and in embryonic stem cells^45^,^46^,^47^,^48^. Taylor *et al*. reported genome-wide mapping of DNA hydroxymethylation in OA chondrocytes^49^. DNA methylation at promoter regions is associated with gene silencing^50^. In OA chondrocytes, higher global 5hmC level was observed, and also 5hmC level was increased particularly in the MMP1 and MMP3 promoter^28^. In our study, bisulfite sequencing results suggest that TWIST1 does not lead to demethylation, but 5hmC levels were increased by TWIST1 in the MMP3 promoter (Fig. 3B,D). This suggests that 5hmC gain may be important but demethylation is not essential for transcriptional regulation of MMP3 by TWIST1. Taylor *et al*. identified 5hmC distribution to be enriched in the regulatory regions of genes preceding the transcription start site (TSS), as well as in the gene bodies during chondrogenic differentiation in ATDC5 cells^51^. To clarify 5mC and 5hmC states in the MMP3 promoter, additional TAB-seq (Tet-assisted bisulfite sequencing) analysis should be performed in future studies. Genome-wide mapping results showed a gain in 5hmC in the gene bodies of some OA susceptibility genes^49^. In our results, 5hmC gain in MMP3 gene body is unknown, but MMP3 induction by TWIST1 was suppressed in human chondrocytes with TET1 knock-down (Fig. 6A) and in Tet triple KO fibroblasts (Fig. 6B), and this results strongly suggests that TET catalyzed enrichment of 5hmC is critical for MMP3 regulation by TWIST1. Previous studies described that globally increased 5hmC level were observed, but TET1 was down-regulated, and there were no significant changes in the expression of TET2 and TET3 in OA chondrocytes^28^. We also confirmed 5hmC enrichment in OA cartilage by immunohistochemistry (Fig. 3A), but in our case, the mRNA levels of TET1 were only mildly increased in human OA knee cartilages compared to normal knee cartilages (Figure S1). These differences of TET1 expression might be caused by the difference between chondrocytes and cartilage tissues, but we also confirmed by immunohistochemistry that TET1 is highly expressed in OA cluster cells (Fig. 5E). TWIST1 dramatically induced TET1 expression in human chondrocytes and TC28 cells, so our results imply that the 5hmC gain is mediated by TWIST1. Two recent reports described that the inflammatory cytokines IL-1β, TNFα or oncostatin M suppressed TET1 gene expression in chondrocytes^28^,^52^, but our results showed TET1 expression didn’t change after IL-1β and TNFα stimulation (Figure S1). These cytokines up-regulated TWIST1 expression (Fig. 1C,D), and TWIST1 overexpression induced TET1 gene expression in human chondrocytes (Figs 4D, 5C and D). Thus, only cytokine stimulation might not be sufficient to regulate TET1 expression through TWIST1 up-regulation. We confirmed TET1 had a critical role in TWIST1 dependent MMP3 upregulation in human primary chondrocytes with siRNA-mediated TET1 knock down (Fig. 6A), and in fibroblasts with TET triple knock-out (Fig. 6B). Therefore, TET expression change through TWIST1 could be an important mechanism for the development of OA.

We observed 5hmC enrichment in the MMP3 promoter (Fig. 3D), but not in the MMP1 promoter (data not shown). Wu *et al*. performed denovo motif discovery analysis, and identified a CpG-rich sequence bound TET1^53^. The MMP3 promoter has two potential TET1 binding sequences (5′-CCGCAGC-3′ and 5′-GCGCAGC-3′) in reverse complement, but the MMP1 promoter has no TET1 binding sequences. These differences between MMP1 and MMP3 promoters are one potential reason that TET1 induces only MMP3 expression.

## Conclusions

This study provides the first evidence that increased expression of TWIST1 is a novel feature of OA-affected cartilage, and TWIST1 promotes catabolic reactions by inducing MMP3 expression through 5hmC gain in MMP3 promoter via regulation of TET1. TWIST1 may be one of core regulators of DNA hydroxymethylation in chondrocytes. Thus, TWIST1 is a promising transcription factor to reveal the detailed epigenetic mechanism in chondrocytes. Dysregulated TWIST1 may be an important factor in OA pathogenesis and a novel treatment target for OA.

## Methods

### Cartilage tissue procurement, cell isolation and culture

The study was conducted in accordance with the Declaration of Helsinki, and the Scripps Institutional Review Board approved the protocol 09–5162 and informed consent form prior to study initiation. Informed consent was obtained from all patients prior the study. Normal human knee joints were procured at autopsy from 7 donors (mean ± SD age 35.1 ± 11.8 years). OA-affected cartilage was collected at the time of total knee arthroplasty from 8 donors with knee OA (mean ± SD age 69.7 ± 5.0 years). We scored articular cartilage by dividing it into 39 regions based on the International Cartilage Repair Society knee map, and evaluated the cartilage macroscopically using a modified Outerbridge scoring system. Each area was scored on a scale of 1–4, where 1 = intact surface, 2 = fibrillation, 3 = fissuring, and 4 = erosion. The total knee cartilage score could thus ranges from 39 (normal) to 156 (maximum severity). Total knee cartilage scores were then translated into grades 0–IV, where grade 0 = normal (total score 39), grade I = minimal change (total score 40–58), grade II = mild change (total score 59–78), grade III = moderate change (total score 79–97), and grade IV = severe change (total score ≥ 98). We considered grade I and II as normal, and grade III and IV as OA.

Chondrocytes were isolated by collagenase digestion of cartilage and cultured in monolayer as described^54^. First passage cells were used in the experiments.

TC28 cells (immortalized human primary juvenile costal chondrocytes) were obtained from the American Type Culture Collection (ATCC). These cells were propagated as monolayer cultures in Dulbecco’s modified Eagle’s medium (DMEM). All media were supplemented with 10% heat-inactivated FBS, 100 units/mL penicillin, and 100 mg/mL streptomycin. The cells were maintained at 37 °C in a humidified atmosphere with 5% CO_2_.

### RNA isolation and quantitative real time-PCR analysis

Total RNA was isolated from cartilage tissue or cells by extracting the homogenate in TRIzol (Life Technologies). Complementary DNA (cDNA) was synthesized using a PrimeScript RT Reagent Kit (Takara Bio). Quantitative polymerase chain reaction (qPCR) was performed using a LightCycler 96 (Roche). TaqMan Gene Expression Assay probes for TWIST1 (Hs00361186_m1), MMP1 (Hs00899658_m1), MMP2 (Hs01548727_m1), MMP3 (Hs00968305_m1), MMP8 (Hs01029057_m1), MMP9 (Hs00234579_m1), MMP10 (Hs00233987_m1), MMP11 (Hs00968295_m1), MMP12 (Hs00159178_m1), MMP13 (Hs00233992_m1), TET1 (Hs00286756_m1), TET2 (Hs00325999_m1) and TET3 (Hs00379125_m1) according to the instructions of the manufacturer (Applied Biosystems). Gene expression levels of GAPDH (Hs02758991_g1) were assessed as an internal control.

### Western blot analysis

TC28 cells and chondrocytes were seeded in 24-well dishes at a density of 1 × 10^4^ cells/dish 24 hours before transfection. Whole cell lysates were prepared in a lysis buffer (50 mM Tris-HCl (pH 7.4), 150 mM NaCl, 1% Triton X-100) containing a protease/phosphatase inhibitor cocktail (Cell Signaling Technology) at the indicated time points. Proteins were electrophoresed on 4–20% SDS polyacrylamide gels and were transferred to nitro cellulose membranes (Bio-Rad). Blots were blocked with Odyssey Blocking Buffer (LI-COR) at room temperature for 30 minutes. The primary antibodies used were: mouse anti-GAPDH monoclonal antibody (mAb) (Ambion), mouse anti-TWIST1 mAb (Sigma-Aldrich), rabbit anti-MMP3 mAb (Cell Signaling Technology), rabbit anti-Myc-Tag polyclonal antibody (pAb) (Cell Signaling Technology), mouse anti-TET1 mAb (GeneTex), rabbit anti-TET2 pAb (GeneTex), rabbit anti-TET3 pAb (GeneTex). The secondary antibodies (goat anti-rabbit – IRDye 800 (1:5000 dilution) and goat anti mouse – IRDye 680 (1:10,000 dilution)) were applied. Bands on the blots were visualized using LiCor Odyssey.

### Plasmid

TWIST1 (Myc-DDK-tagged)-Human twist 1 was obtained from Origene. For making piggyBac Myc-TWIST1 transposon vector, the human TWIST1 coding region was obtained from the cDNA with KOD Hot Start DNA Polymerase (Novagen), and subcloned into a piggyBac vector. The primer sequences to clone the coding region of TWIST1 were as follows: Forward primer: 5′-ATGATGCAGGACGTGTCCAGCTCGCC-3′; Reverse primer: 5′-CTAGTGGGACGCGGACATGGACCAGGC-3′. To induce DNA binding defective mutant of TWIST1, basic helix-loop-helix region deleted TWIST1 open reading frame (ORF) over expression vector (piggyBac TWIST1-ΔbHLH vector) was constructed^55^.

### Adenovirus

TWIST1-adenovirus (Ad-TWIST1) (SignaGen Laboratories) and eGFP-adenovirus (Ad-GFP) (VECTOR Biolabs) were used in this study. TC28 cells were infected with adenovirus at a multiplicity of infections (MOI) of 100 plaque forming units (PFU)/cell, and human chondrocytes were infected at 1000 MOI.

### Bisulfite genomic sequencing analysis

To study DNA methylation status by methylation-specific PCR in TC28 cells after TWIST1 over expression, the DNA was treated with bisulfite using the EZ DNA methylation-Gold KIT (ZYMO Research Corp). After PCR amplification, the target DNA fragment was subcloned into pCR4-TOPO (Life Technologies), and sequenced.

### Analyzing and quantitating 5hmC in MMP3 promoter

5hmC levels were detected using EpiMark 5hmC and 5mC Analysis Kit (New England Biolabs). TC28 cells were seeded in 6-well plates at a density of 2 × 10^5^ cells/well 24 hours before Ad-GFP or Ad-TWIST1 infection. Genomic DNA was extracted from the cells 2 days after virus infection by using PureLink Genomic DNA Mini Kit. Genomic DNA (10 μg) was treated with 30 units of T4 β-glucosyltransferase (T4-BGT) at 37 °C for 18 hours. 100 units of MspI or 50 units of HpaII was added to the glucosylated DNA, and incubated the sample at 37 °C for 12 hours. Proteinase K treatment was done at 40° for 30 minutes and inactivated at 95° for 10 minutes. The percentages of 5hmC were calculated EpiMark comparative Ct method. The sequences of primers for MMP3 promoter were 5′-GGAGGGGAAAAGGTTGAAAG-3′ (forward) and 5′-CCACGTAGCTGCTCCATAAATAG-3′ (reverse).

### Immunohistochemistry

Immunohistochemistry studies were performed to investigate the expression pattern of TWIST1 in human cartilage tissue. The tissue sections were incubated with 10% trypsin at 37 °C for 60 minutes for antigen retrieval. After washing with phosphate buffered saline, the sections were blocked with 10% normal goat serum for 60 minutes at room temperature. Rabbit anti-human TWIST1 polyclonal antibody (Cell Signaling Technology), rabbit polyclonal anti-MMP3 antibody (Cell Signaling Technology), rabbit polyclonal anti-5hmC antibody (Active Motif) and mouse anti-TET1 antibody (GeneTex) were applied and incubated overnight at 4 °C. The sections were incubated with secondary antibody, substrate and hematoxylin were performed.

### Fibroblasts from Tet1, 2 and 3 triple knock out embryonic stem cells

Mesenchymal cells were produced through induction of embryoid bodies (EBs)^56^. Undifferentiated Tet triple knock out (TKO) embryonic stem (ES) cells were dissociated into single cell suspension by treatment with 0.25% trypsin (Invitrogen) and 0.02% EDTA (SIGMA-Aldrich) in PBS (trypsin-EDTA), resuspended in 10% FBS-DMEM (Invitrogen), and then transferred to suspension culture on Petri dishes. After 3 days of EB formation, cell aggregates were cultured on the gelatin-coated dishes with 10% FBS-DMEM supplemented with 10–6 M all-trans retinoic acid (RA, SIGMA-Aldrich). After 8 days, the outgrowths were dissociated, filtered by 40-μm meshes (BD Biosciences Discovery Labware, MA, USA) and cultured briefly (~1 hour) on the gelatin-coated culture dishes to select only the fibroblast cells. Then, the attached cells were cultured in 10% FBS-αMEM supplemented with 10 ng/ml bFGF (Upstate Biotechnology, Lake Placid, NY, USA) until confluence.

### Statistical analysis

Data are reported as mean ± SEM of the indicated number of independent experiments. Some experiments were conducted with primary chondrocytes samples from three different donors, each performed in triplicate. Statistically differences were determined by t-tests or one-way ANOVA with post hoc comparisons using Tukey’s test. P values less than 0.05 were considered significant.

## Additional Information

**How to cite this article**: Hasei, J. *et al*. TWIST1 induces MMP3 expression through up-regulating DNA hydroxymethylation and promotes catabolic responses in human chondrocytes. *Sci. Rep.* **7**, 42990; doi: 10.1038/srep42990 (2017).

**Publisher's note:** Springer Nature remains neutral with regard to jurisdictional claims in published maps and institutional affiliations.

## Supplementary Material

Supplementary Figure
