# Supplementary material for: TWIST1 induces MMP3 expression through up-regulating DNA hydroxymethylation and promotes catabolic responses in human chondrocytes
Source: Sci Rep. 2017 Feb 21;7:42990. doi: 10.1038/srep42990 (PMC5318945; doi:10.1038/srep42990)
Supplement: Supplementary Figure [file srep42990-s1.pdf]

Supplementary Figure

Joe Hasei[1], Takeshi Teramura[1, 2], Toshiyuki Takehara[2], Yuta Onodera[2], Takuro Horii[3], Merissa Olmer[1], Izuho Hatada[3], Kanji Fukuda[2, 4], Toshifumi Ozaki[5], Martin K. Lotz[1] and Hiroshi Asahara[1, 6]
[1]Department of Molecular and Experimental Medicine, The Scripps Research Institute, La Jolla, CA, USA.
[2]Division of Cell Biology for Regenerative Medicine, Institute of Advanced Clinical Medicine, Kindai University, Faculty of Medicine, Osaka, Japan.
[3]Laboratory of Genome Science, Biosignal Genome Resource Center, Institute for Molecular and Cellular Regulation, Gunma University, Gunma, Japan.
[4]Department of Rehabilitation Medicine, Kindai University, Faculty of Medicine, Osaka, Japan.
[5]Science of Functional Recovery and Reconstruction, Okayama University Graduate School of Medicine, Dentistry and Pharmaceutical Sciences, Okayama, Japan
[6]Department of Systems BioMedicine, Graduate School of Medical and Dental Sciences, Tokyo Medical and Dental University, Tokyo, Japan.

TWIST1 induces MMP3 expression through up-regulating DNA hydroxymethylation and promotes catabolic responses in human chondrocytes

Supplementary Figure S1
The TETs expression in normal and OA-affected human cartilage tissue.

Supplementary Figure S2
The TETs expression change after IL-1β or TNFα stimulation in human chondrocyte.

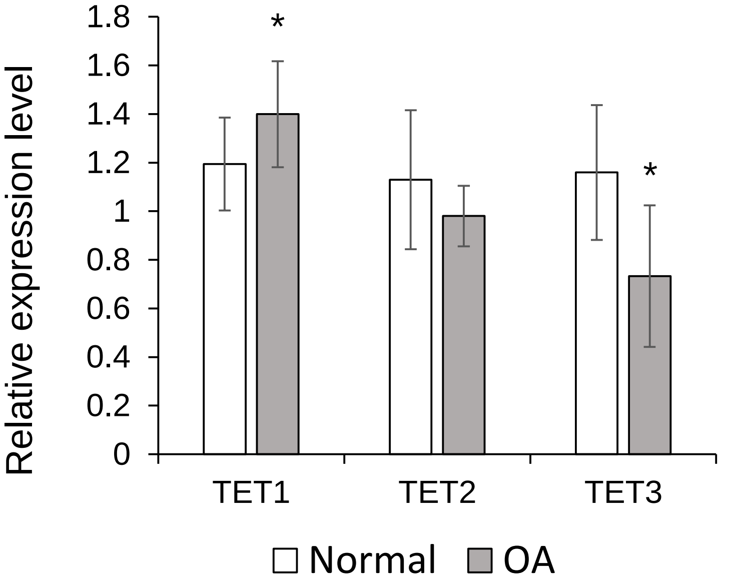

Supplementary Figure S1

The TETs expression in normal and OA-affected human cartilage tissue.

TWIST1 gene expression in cartilage tissues from 6 normal donors and 6 OA donors were analyzed by real-time PCR. TETs gene expression levels in OA cartilage tissues are relative to normal cartilage tissues. Values are the mean ± SEM ratio.

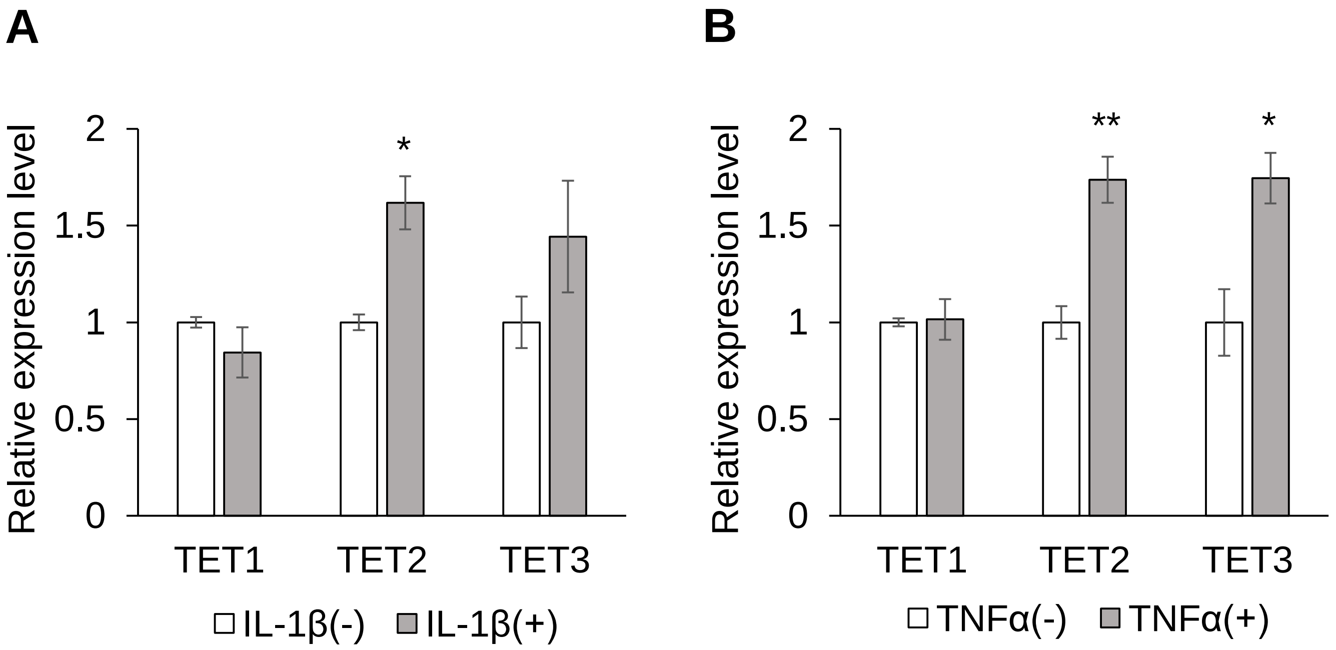

Supplementary Figure S2
The TETs expression change after IL-1β or TNFα stimulation in human chondrocytes. A, The relative TETs expression after IL-1β stimulation. B, The relative TETs expression after TNFα stimulation. Values are the mean ± SEM ratio. *= $P < 0.05$, **= $P < 0.01$.

Supplementary Materials and Methods

Quantitative real-time PCR analysis.

To confirm the TETs gene expression in human normal and OA chondrocytes, the chondrocytes were seeded on 6-well plates 24 hours before Ad-GFP or Ad-TWIST1 infection at an MOI of 100 PFUs/cell. 72 hours after viral infection, the TETs gene expressions were analyzed by real-time qRT-PCR. To confirm the TETs expression change after IL-1β or TNFα stimulation in human chondrocytes, the chondrocytes were seeded on 6-well plates 24 hours before IL-1β or TNFα stimulation. After 12 hours cytokine treatment, TETs gene expression were analyzed by real-time qRT-PCR.
